# The indication and effectiveness of low-dose erythromycin therapy in pediatric patients with bronchial asthma*

**DOI:** 10.1111/j.1399-3038.2009.00941.x

**Published:** 2010-05

**Authors:** Seigo Korematsu, Kyoko Yamamoto, Tomokazu Nagakura, Hiroaki Miyahara, Naho Okazaki, Kensuke Akiyoshi, Tomoki Maeda, So-ichi Suenobu, Tatsuro Izumi

**Affiliations:** Department of Pediatrics and Child Neurology, Faculty of Medicine, Oita UniversityOita, Japan

**Keywords:** bronchial asthma, interleukin 8, low-dose erythromycin, theophylline, vascular endothelial growth factor

## Abstract

Korematsu S, Yamamoto K, Nagakura T, Miyahara H, Okazaki N, Akiyoshi K, Maeda T, Suenobu S-i, Izumi T. The indication and effectiveness of low-dose erythromycin therapy in pediatric patients with bronchial asthma. Pediatr Allergy Immunol 2010: 21: 489–492. © 2010 John Wiley & Sons A/S

To elucidate the mechanisms of intractable pediatric bronchial asthma and the indication of low-dose erythromycin (EM) therapy, the serum chemokine levels of and the angiogenic factor were evaluated in 55 pediatric patients with bronchial asthma; 7.4 ± 3.5 yr old, who had been treated with inhaled steroid, leukotriene receptor antagonist, theophylline and others for more than a year. Both the levels of interleukin (IL) 8 (p = 0.036) and vascular endothelial growth factor (VEGF) (p = 0.005) were higher in patients with severe type than those of patients with the milder type, while other chemokine levels such as serum eotaxin and MCP1 did not show the correlation with the severity of bronchial asthma. Induction of therapy with low-dose EM induced improvement of the clinical symptoms in patients with severe type and decrease of their serum chemokine levels: IL8; from 736 ± 88 to 75 ± 85 pg/ml (p < 0.0005), and VEGF; from 352.0 ± 160.5 to 132.2 ± 59.9 pg/ml (p = 0.021) within the next 6 months. Moreover, low-dose EM resulted in a decreased daily peak-trough fluctuation rate of the serum theophylline concentration; (*C*_max_− *C*_min_)/*C*_min_, from 1.3 ± 0.5 to 0.5 ± 0.3, which led to the maintenance of effective serum levels. These results indicated that IL8 and VEGF affect the severity of standard therapies resistance intractable bronchial asthma. Through the suppression of these chemokines and maintenance of effective theophylline levels, low-dose EM therapy improves the symptoms of bronchial asthma.

Low-dose erythromycin (EM) has been administered to patients with chronic respiratory diseases, such as diffuse panbronchiolitis and chronic pansinusitis to suppress the neutrophilic inflammation process of interleukin (IL) 8 (1). Regarding the bronchial asthma, low-dose EM therapy has not been indicated for patients with bronchial asthma because the essential mechanism of bronchial asthma is considered as an eosinophilic inflammation; if anything, the co-administration of EM and theophylline has been avoided because EM inhibits the theophylline metabolism (2). Recently, several reports (3, 4) showed the effectiveness of low dose macrolide antibiotics in some patients with bronchial asthma, however, the indication of this therapy and the influence to theophylline metabolism have not been examined especially in pediatric patients.

In this study, to elucidate the effectiveness and the indication of low dose EM therapy, serum chemokine characterization and the theophylline metabolism in pediatric patients with bronchial asthma were evaluated.

## Patients

Fifty-five pediatric patients with bronchial asthma; 7.4 ± 3.5 yr old and 10 age-matched non-allergic non-febrile disease controls were included in this study after obtaining the informed consent from both the children and their parents. The diagnosis and severity of bronchial asthma were evaluated using the global initiative for asthma (GINA) guidelines (6); a positive specific IgE to such environmental allergens as house dust mites, Japanese cedar and others, a peripheral blood eosinophilia and an obstructive respiratory function pattern. These patients had been administered inhaled steroids (fluticasone; 200–400 μg/day), leukotriene receptor antagonists, theophylline and others for more than a year. Through those standard therapies, most patients with bronchial asthma improved their clinical symptoms, however, only four patients repeatedly developed moderate-severe asthma attack. Regarding the clinical severity of asthma, four patients showed severe-intractable type, 23 patients showed moderate-type and 28 patients showed mild-type.

## Methods

Patient blood samples were collected during a stable state without any infections or fever at least the previous 2 wk. Infections such as *Mycoplasma*, *Chlamydia* and respiratory syncytial virus were ruled out in all asthma patients by their serum antibody titers. The serum levels of IL8; neutrophilic chemokine, eotaxin; eosinophilic chemokine, monocyte chemoattractant protein (MCP) 1; monocytic chemokine, vascular endothelial growth factor (VEGF) were examined using enzyme linked immunosorbent assays (ELISA) kit (Amersham Pharmacia Biotech, Piscataway, NJ, USA). The detection limits were 5.0, 5.0, 25.0 and 5.0 pg/ml, respectively.

Low-dose EM (10 mg/kg/day divided twice) was added on their current medications such as inhaled steroid, leukotriene receptor antagonist and theophylline for four severe-type patients; two of four patients also suffered from allergic pansinusitis. In addition, their clinical symptoms and serum chemokines levels were compared before and after 6 months of low-dose EM therapy.

Moreover, three of four patients with severe-type asthma had been administered theophylline 16 mg/kg/day divided twice for more than a year. Before and 6 months after the co-administration of low-dose EM and theophylline, theophylline clearance, half-time and AUC (area under the curve) were calculated using the THEOPREDICT-III (Nikken Chemicals Co. Ltd, Tokyo, Japan and Mitsubishi Pharma Co. Ltd, Tokyo, Japan) based on 4–6 samples of the serum theophylline concentrations such as trough levels, and 4 and 6 h after administration. The daily peak-trough fluctuation rate of theophylline level was calculated using the formula; (*C*_max_− *C*_min_)/*C*_min_.

A statistical examination was performed using the Kruskal–Wallis test and the Wilcoxon signed rank test.

## Results

As described in Table 1, the serum IL8 increased according to asthma severity; healthy controls, mild-type, moderate-type and severe-type were 22.5 ± 11.5, 34.9 ± 61.3, 82.0 ± 145.1, 735.5 ± 88.0 pg/ml, respectively (p = 0.036). The serum VEGF also increased according to severity; 35.1 ± 78.5, 78.3 ± 68.5, 149.1 ± 86.6, 352.0 ± 160.5 pg/ml, respectively (p = 0.005).

**Table 1:** The Serum chemokine characterizations in patients with asthma

				Severe (4)	
Severity (n)	Control (10)	Mild (28)	Moderate (23)	Before EM	After EM
IL8 (pg/ml)	22.5 ± 11.5	34.9 ± 61.3	82.0 ± 145.1	735.5 ± 88.0	74.8 ± 84.7
	p = 0.036			p < 0.0005	
Eotaxin (pg/ml)	36.2 ± 8.3	67.1 ± 38.2	51.0 ± 16.5	49.7 ± 23.5	45.0 ± 28.8
	p = 0.358			p = 0.778	
MCP1 (pg/ml)	390 ± 113	970 ± 546	850 ± 445	1869 ± 382	1306 ± 21
	p = 0.078			p = 0.043	
VEGF (pg/ml)	35.1 ± 78.5	78.3 ± 68.5	149.1 ± 86.2	352.0 ± 160.5	132.2 ± 59.9
	p = 0.005			p = 0.021	

After the administration of low-dose EM to four severe-type patients, their clinical symptoms improved (Table 2) and the levels of both IL8; from 735.5 ± 88.0 to 74.8 ± 84.7 pg/ml (p < 0.0005) and VEGF; from 352.0 ± 160.5 to 132.2 ± 59.9 pg/ml (p = 0.021) decreased (Table 1). Both of the serum eotaxin levels and serum MCP1 levels were not correlated with the severity and EM administration.

**Table 2:** Changes of clinical symptoms and theophylline metabolism in patients with severe-type asthma

Case	Age (yr)	Sex	Before EM	6 months after EM
1	2	F	Severe attack:1/1 month	Severe attack:1/6 months
2	7	M	Severe attack:1/2 months %FEV_1.0_ 120%	Moderate attack:1/6 months %FEV_1.0_ 145%
3	9	M	Moderate attack:1/2 wk %FEV_1.0_ 55%	Moderate attack:1/2 months %FEV_1.0_ 77%
4	13	M	Moderate attack:1/1 month %FEV_1.0_ 75%	Moderate attack:1/6 months %FEV_1.0_ 83%

TDM\case	1	2	3	Mean
CL (l/kg/h)	0.094/0.077	0.093/0.055	0.092/0.054	0.093/0.062
*t*_1/2_ (h)	5.4/6.6	6.2/12.7	7.3/10.7	6.3/9.9
AUC (μg/h/ml)	99/120	247/418	160/247	169/262
*C*_min_ (μg/ml)	3.0/8.2	7.8/15.1	3.5/9.2	4.8/10.8
*C*_max_ (μg/ml)	6.5/14.7	14.9/18.0	10.3/14.2	10.6/15.6
*C*_max_− *C*_min_/*C*_min_	1.2/0.8	0.9/0.2	1.9/0.5	1.3/0.5 (before/6 months after EM)

EM, erythromycin; %FEV_1.0_, % predict value of 1 second forced expiratory volume; CL, clearance; *t*_l/2_, half-time; AUC, area under the curve; C, concentration.

After the co-administration of EM and theophylline for three of four severe-type patients, the theophylline clearance decreased from 0.093 ± 0.001 to 0.062 ± 0.013 l/kg/h, and the half-time increased from 6.3 ± 1.0 to 9.9 ± 3.3 h and the AUC (area under the curve) increased from 168.7 ± 74.4 to 261.7 ± 149.5 mg/CL × Wt. Such an altered theophylline metabolism resulted in a decreased daily peak-trough fluctuation rate of the serum theophylline concentration; (*C*_max_− *C*_min_)/*C*_min_, from 1.3 ± 0.5 to 0.5 ± 0.3 (Table 2).

## Discussion

Long-term low dose EM therapy has been introduced for the treatment of diffuse panbronchiolitis and chronic pansinusitis (1). Low-dose EM does not act as a bactericide but it inhibits the IL8 production by the recruited neutrophils (1). Although bronchial asthma is an essentially caused by eosinophilic inflammation (6), an association between asthma and neutrophilc inflammation has been described in recent years. Norzila MZ et al. (5), Marquet C et al. (7), and Kikuchi I et al. (8) showed the recruitment of IL8 produced by neutrophils in patients with acute asthma attack. Gounni AS et al.(9) described that not only mast cells but also neutrophils of patients with asthma express the high-affinity receptor for IgE, and the binding of IgE to the receptor leads to IL8 production from the neutrophils.

On the other hand, VEGF; one of the angiogenic factors, is produced by neutrophil (10). It has also been reported to be associated with airway re-modeling; which is one of the exacerbation factors of asthma (11). The effect of 14-membered ring macrolide antibiotics on the production of VEGF still remains unclear, however, the inhibition of VEGF derived from lung cancer cells by roxithromycin has been reported (12). EM might also inhibit VEGF in patients with bronchial asthma.

Because of their immaturity regarding expiratory procedures, examinations using sputum tend to be difficult to perform in pediatric patients. Therefore, serum chemokine examinations were instead performed in this study.

Our results showed the severity of bronchial asthma and the serum levels of IL8 and VEGF to demonstrate a positive correlation; yet, there was no significant difference was observed in terms of both serum eosinophilic chemokine eotaxin and monocytic chemokine MCP1. Based on the findings of eotaxin, eosinophilic inflammation might already be inhibited by such long-term medications as inhaled steroids, leukotriene receptor antagonist, theophylline and others. And thereafter, both IL8 and VEGF levels in patients with severe-type were decreased after 6 months of the low-dose EM treatment regimen, and followed by the improvement in clinical symptoms of bronchial asthma.

These results suggest that the neutrophilic inflammation thus plays a role in the severity of standard therapies resistance bronchial asthma. Because only two of four patients with severe type asthma suffered from allergic pansinusitis, the effectiveness of low-dose EM therapy might not only be due to the improvement of nasal symptoms (13). Although infections of *Mycoplasma*, *Chlamydia* and respiratory syncytial virus, which cause acute neutrophilic inflammation followed by transient wheezing (14), were ruled out in these patients by their serum antibody titers, some kind of chronic respiratory infections might be persistent in intractable patients with asthma.

Furthermore, an inhibition in the theophylline metabolism, which was considered to be a ‘side effect’ of EM, also demonstrated a beneficial effect. In severe-type patients, it was found to be difficult to maintain the serum theophylline levels within the therapeutic range; it therefore helped to decrease the daily fluctuations of the levels. Since an increased dose of theophylline alone would not help to induce an altered their daily fluctuation rates, this unexpected discovery might thus be the second important mechanism of low-dose EM therapy. Although theophylline should be administered carefully because it lowers the seizure threshold, especially in infantile and/or febrile patients (15), it might be still necessary for intractable asthma patients.

In this study, only four patients with severe-type were examined because most pediatric patients have become easier to control with standard therapies such as inhaled steroids, leukotriene receptor antagonist, theophylline and others. For this limitation, a further large scale double blind placebo control study should be needed next. In patients with still intractable asthma, low-dose EM therapy might be effective through the suppression of IL8 and VEGF and maintenance of effective theophylline levels.

## Abbreviations:

AUC: area under the curve
ELISA: enzyme linked immunosorbent assays
EM: erythromycin
GINA: global initiative for asthma
IL: interleukin
MCP1: monocyte chemotactic protein-1
*t*_1/2_: half time
VEGF: vascular endothelial growth factor
